# Impact of *TP53* mutations in acute myeloid leukemia patients treated with azacitidine

**DOI:** 10.1371/journal.pone.0238795

**Published:** 2020-10-01

**Authors:** Pierre Bories, Naïs Prade, Stéphanie Lagarde, Bastien Cabarrou, Laetitia Largeaud, Julien Plenecassagnes, Isabelle Luquet, Véronique De Mas, Thomas Filleron, Manon Cassou, Audrey Sarry, Luc-Matthieu Fornecker, Célestine Simand, Sarah Bertoli, Christian Recher, Eric Delabesse

**Affiliations:** 1 Laboratoire d'Hématologie, Centre Hospitalier Universitaire de Toulouse, Institut Universitaire du Cancer de Toulouse Oncopole, Toulouse, France; 2 Service d'Hématologie, Centre Hospitalier Universitaire de Toulouse, Institut Universitaire du Cancer de Toulouse Oncopole, Toulouse, France; 3 Réseau Onco-occitanie, Institut Universitaire du Cancer de Toulouse Oncopole, Toulouse, France; 4 Unité de biostatistique, Institut Claudius Régaud, Institut Universitaire du Cancer de Toulouse Oncopole, Toulouse, France; 5 Unité de bioinformatique, Institut Claudius Régaud, Institut Universitaire du Cancer de Toulouse Oncopole, Toulouse, France; 6 Service d'Onco-Hématologie, Centre Hospitalier Universitaire de Strasbourg, Strasbourg, France; European Institute of Oncology, ITALY

## Abstract

Hypomethylating agents are a classical frontline low-intensity therapy for older patients with acute myeloid leukemia. Recently, *TP53* gene mutations have been described as a potential predictive biomarker of better outcome in patients treated with a ten-day decitabine regimen., However, functional characteristics of TP53 mutant are heterogeneous, as reflected in multiple functional TP53 classifications and their impact in patients treated with azacitidine is less clear. We analyzed the therapeutic course and outcome of 279 patients treated with azacitidine between 2007 and 2016, prospectively enrolled in our regional healthcare network. By screening 224 of them, we detected *TP53* mutations in 55 patients (24.6%), including 53 patients (96.4%) harboring high-risk cytogenetics. The identification of any *TP53* mutation was associated with worse overall survival but not with response to azacitidine in the whole cohort and in the subgroup of patients with adverse karyotype. Stratification of patients according to three recent validated functional classifications did not allow the identification of *TP53* mutated patients who could benefit from azacitidine. Systematic TP53 mutant classification will deserve further exploration in the setting of patients treated with conventional therapy and in the emerging field of therapies targeting TP53 pathway.

## Introduction

With little improvement in their overall survival (OS) over the last decade, older patients with acute myeloid leukemia (AML) still harbor dismal prognosis [1, 2]. Validated therapeutic options are currently limited. Patient selection for intensive versus low-intensity therapy remains controversial [3, 4] and inter-physician practice variations are frequent, which underscores the uncertainty on the optimal strategy for any elderly AML patient [5].

Although azacitidine failed to demonstrate its superiority in the AZA-AML-001 trial compared to intensive chemotherapy (IC) for patients older than 65 years with non-proliferative AML [6], several studies have found that patients with adverse cytogenetic risk myelodysplastic syndrome (MDS) or AML treated with hypomethylating agents (HMA) may obtain similar or even higher response rates than patients with intermediate-risk cytogenetics [7–9]. The prevalence of *TP53* mutations is typically extremely high (up to 50–70% in AML with complex karyotype [10–12]) in this population and the efficacy of HMA may reflect a TP53-independent mechanism of action. Alteration of TP53 functions is a well-known negative prognostic factor for MDS and AML patients treated with conventional chemotherapy [10, 13] and allogeneic stem cell transplantation [14–16], which has justified investigation of alternative TP53-independent therapy such as HMA. In preclinical studies, primary fibroblastic and for *TP53-*deficient neoplastic cells exhibit hypersensitivity to decitabine treatment compared to wild type cells, through apoptotic response [17, 18], illustrating a previously described concept of sensitization to apoptosis by the absence of *TP53* [19, 20] and extending this concept to HMA. A single-institution trial has described a protective effect of *TP53* mutations in AML patients treated frontline with an intensified regimen of decitabine [21]. However, only 21 patients (18%) harboring a *TP53* mutation were included in this trial, rendering necessary a confirmation of these interesting results. More recently, the pivotal AZA-AML-001 phase 3 trial described no significant association between *TP53* mutations and outcome of AML patients treated with azacitidine while *TP53* mutations remained associated with shorter OS in the conventional care comparator arm [22].

Although mutations in *TP53* have traditionally been considered functionally equivalent leading to a lack of function due the loss of the DNA-binding domain or mediated by a dominant-negative effect on the remaining functional wild-type allele, recently, some TP53 mutants were shown to display a gain of function (GOF) independent of wild-type *TP53* (TP53^wt^) function [23]. Several classifications of *TP53* mutants have been proposed with a correlation to the patient outcome in solid tumors and diffuse large B cell lymphoma [24–26]. Their clinical usefulness has never been questioned in AML patients, although it might be highly important in the context of novel therapy targeting *TP53* and/or *MDM2*.

We took advantage of our extensively analyzed prospective regional AML registry [1, 27, 28] to investigate the impact of *TP53* mutations in a very large cohort of AML patients treated frontline with azacitidine. We further assessed the usefulness of *TP53* mutation classifications as a biomarker with the aim to eventually identify a sub-group of patients with *TP53* mutations who might specifically benefit from azacitidine.

## Patients and methods

### Regional cancer network ONCOMIP registry

AML patients (excluding M3) treated frontline with azacitidine were enrolled in the regional cancer network ONCOMIP registry between 2007 and 2016 [1, 27, 28] (Toulouse AML database). Patient’s bone marrow samples were obtained following standard ethical procedures (Helsinki principles), after informed written consent, and stored at the HIMIP collection. According to the French law, the HIMIP collection was declared to the Ministry of Higher Education and Research (DC 2008–307 collection 1). The French Commission Nationale de l’Informatique et des Libertés (CNIL) authorised the use of patient data analyzed in our study Cytogenetic risk was assessed according to the MRC classification [29].

### *TP53* next generation sequencing

Genomic DNA (gDNA) was extracted from baseline bone marrow sample using a Qiagen DNA extraction kit (Qiagen). *TP53* status was derived from exome sequencing for 49 patients or using a Next Generation Sequencing multiplex PCR for 179 patients.

Exome capture was performed using Sureselect All-Exome V4 kit (Agilent). Exome libraries were then sequenced using a NextSeq500 sequencer (Illumina) and a SureSelect QXT Reagent kit (paired-end, 150bp, sequencing 2 x 150 cycles).

*TP53* Next Generation Sequencing was performed using a multiplex *TP53* PCR covering the complete coding sequences of exons 4 to 10 (primers listed in S1 Table). *TP53* libraries were then sequenced using a Miseq Reagent kit V2 (paired-end, 150bp, sequencing 2 x 150 cycles) and MiSeq sequencer (Illumina).

Alignment and variant calling were performed using NextGene software (SoftGenetics). *TP53* variants with a variant allele frequency (VAF) higher than 1%, were filtered using the *TP53* International Agency for Research on Cancer R18 database released in April 2016 (IARC) (http://www-p53.iarc.fr/) [30].

### TP53 mutation classifications

Each *TP53* mutation was analyzed according to 3 different classifications. For patients with more than one *TP53* mutation, we selected the mutation with the highest predicted impact [31].

#### (1) Disruption classification [24]

This classification was based on the consequences of the *TP53* mutation on its protein folding, segregating disruptive versus non-disruptive mutations. This clustering was validated as a prognosis factor for OS in a series of head and neck carcinoma patients by Poeta *et al* [24]. It relies on the location of the mutation and the predicted amino acid alterations. Disruptive mutations are composed of (i) stop codons in any region or (ii) non-conservative mutations (i.e. change of category of the mutated amino-acid [non-polar as F, M, W, I, V, L, A and P; polar non charged as C, N, Q, T, Y, S and G; polar negatively charged as D and E and polar positively charged as H, K and R]) inside the key DNA-binding domains (L2–L3 region, corresponding to codons 163 to 195 and 236 to 251). All other mutations are classified as non-disruptive.

#### (2) Evolutionary action TP53 Score classification

Computational approach with the calculation of an evolutionary action score summarizing the phenotype to genotype relationship for each *TP53* missense mutation reliably stratified patients with head and neck cancers or metastatic colon cancers harbouring *TP53* mutations as high or low risk [25, 32]. The score was calculated for each *TP53* missense mutation identified in our cohort [25]. A threshold of 75 derived from the work of Neskey *et al*. was used to separate high risk from low risk *TP53* mutations.

#### (3) Relative fitness score (RFS) classification

Kotler *et al*. used a massively parallel proliferation assay deciphering the sequence to structure and function relationship of *TP53* mutations in human cells, leading to the establishment of a comprehensive catalogue of 9,833 unique *TP53* DNA-binding domain variants (DBD, corresponding to amino-acids 102 to 292) with their functions evaluated in human cells *in vitro* and *in vivo* [33] giving a relative fitness score for each of these TP53 mutant.

### Statistical analyses

The analysis date for the clinical evaluation of the database was June 1, 2018. All the data used for the analyses were deposited in Figshare: https://doi.org/10.6084/m9.figshare.12897077.v1. Clinical response was assessed using ELN criteria [34] after 3 and 6 cycles of azacitidine as indicated. For patient who failed to achieve at least partial clinical remission, we also assessed hematological improvement using the MDS IWG 2006 response criteria [35].

Data were summarized using descriptive statistics. Categorical variables were presented as frequency, percentage and number of missing data. Continuous variables were presented as median, range and number of missing data. Comparisons between groups were performed using the Chi-squared or Fisher’s exact test for categorical variables and the Mann-Whitney test for continuous variables. Duration of response was evaluated in patients achieving a response as defined by complete remission (CR) and complete remission with incomplete hematologic recovery (CRi). It was defined as the time from the date of response to the date of relapse or death from any cause and was estimated using the Kaplan-Meier method. Overall survival (OS) was defined as the time from the date of diagnosis to the date of death from any cause, patients alive were censored at last follow up news. Survival rates were estimated using the Kaplan-Meier method. Univariable and multivariable analyses were performed using the Logrank test and the Cox proportional hazards model; hazard ratios were estimated with their 95% confidence intervals. All tests were two-sided and p-values < 0.05 were considered statistically significant. Statistical analyses were conducted using STATA 13 (StataCorp, Texas, USA) software.

## Results

### Characteristics of patients treated with azacitidine

From January 1^st^ 2007 to December 31^st^ 2016, 279 AML treated frontline with azacitidine were enrolled in the regional cancer network ONCOMIP registry. Patients received a median of 6 cycles of azacitidine (range: 1 to 67) with a median follow up of 66.1 months. Median age was 76 years (range: 45 to 93). AML was secondary to a previous myeloid malignancy in 34% of the cases, MDS in 71 patients (25.4%) or myeloproliferative neoplasms in 24 patients (8.6%). AML was therapy-related in 46 patients (16.5%).

Cytogenetic risk was adverse in 135 patients (49.1%), including 54 patients with complete (-17) or partial (del17p) deletion of chromosome-17, chromosome containing the *TP53* locus (19.4%). *TP53* status was available before azacitidine treatment for 224 patients.

We detected a *TP53* mutation in 55 patients (24.6%) at a VAF threshold of 10% and 64 patients (28.6%, S1 Fig) at a threshold of 1%. The following analysis was done using a VAF threshold of 10%. *TP53* locus was deleted and/or mutated (*TP53* alteration) in 68 patients (30.4%). Patient characteristics according to *TP53* mutational status are summarized in Table 1. Compared to patients without *TP53* mutation, patients harboring a *TP53* mutation presented more often with altered performance status (ECOG score ≥2 in 26.4% *vs*. 42%, respectively, p = 0.037), had a lower baseline median platelet count (74 G/L *vs*. 46 G/L, respectively, p = 0.001) and higher rate of adverse cytogenetics (33.1% *vs*. 96.4%, respectively, p<0.001).

**Table 1:** Patient characteristics according to TP53 status.

	Azacitidine cohort N = 279	*TP53*wt N = 169	*TP53*mut N = 55	*TP53* unknown N = 55	*TP53*wt vs *TP53*mut p value
**Baseline characteristics**					
**Median Age—**years (range)	76 (45–93)	76 (45–90)	75 (50–86)	76 (57–93)	0.089
**Male gender—**n (%)	155 (55.6)	100 (59.2)	29 (52.7)	26 (47.3)	0.401
**AML status—**n (%)					
*De novo*	138 (49.5)	92 (54.4)	26 (47.3)	20 (36.4)	
Secondary to MDS^a^	71 (25.4)	43 (25.4)	9 (16.4)	19 (34.5)	0.084
Secondary to MPN^b^	24 (8.6)	10 (5.9)	7 (12.7)	7 (12.7)	
Therapy related AML	46 (16.5)	24 (14.2)	13 (23.6)	9 (16.4)	
**ECOG performance status—**n (%)					
0–1	168 (69.7)	109 (73.6)	29 (58.0)	30 (69.8)	
2–4	73 (30.3)	39 (26.4)	21(42.0)	13 (30.2)	**0.037**
Unknown	38	21	5	12	
**Charlson score—**n (%)					
0–1	176 (76.5)	103 (73.0)	37 (80.4)	36 (83.7)	
>1	54 (23.5)	38 (27.0)	9 (19.6)	7 (16.3)	0.316
Missing	49	28	9	12	
**Extramedullary disease-**n (%)					
No extramedullary disease	228 (88.7)	140 (88.6)	46 (88.5)	42 (89.4)	0.977
Extramedullary disease	29 (11.3)	18 (11.4)	6 (11.5)	5 (10.6)	
Missing	22	11	3	8	
**Median WBC**^c^ **count (n = 274)—**G/L (range)	2.7 (0.4–271.0)	2.4 (0.7–122.7)	2.3 (0.5–85.0)	3.4 (0.4–271)	0.440
**Median platelet count (n = 274) -**G/L (range)	67 (3–1271)	74 (7–771)	46 (3–1271)	71.5 (5–736)	**0.001**
**Median LDH**^**d**^ **(n = 260)—**U/L (range)	540.5 (135–3525)	502 (135–3525)	569.5 (163–3175)	662.5 (168–3503)	0.149
**Median % BM blast count** (n = 272)**–**(range)	33 (0–85)	35 (0–83)	29.5 (9–85)	28 (2–78)	0.075
**Albumin—n (%)**					
Normal	164 (81.2)	106 (84.8)	32 (74.4)	26 (76.5)	0.125
<Normal	38 (18.8)	19 (15.2)	11 (25.6)	8 (23.5)	
Missing	77	44	12	21	
**Cytogenetics (MRC**^e^**)—**n (%)					
Non adverse	140 (50.9)	113 (66.9)	2 (3.6)	25 (49.0)	**<0.001**
Adverse	135 (49.1)	56 (33.1)	53(96.4)	26 (51.0)	
Unknown	4	0	0	4	
**Monosomal karyotype—**n (%)	66 (24.6)	14 (8.4)	37 (67.3)	15 (31.9)	**<0.001**
**Del17p or monosomy 17**- n (%)	54 (19.4)	13 (7.7)	32 (58.2)	9 (16.4)	**<0.001**
**Outcome**					
**Median number of azacitidine cycles** n(range)	6 (1–67)	8 (1–67)	5 (1–22)	6 (1–26)	**<0.001**
**Response**					**0.502**
CR^f^/CRi^g^ –n(%)	54 (19.4)	30 (17.8)	12 (21.8)	12 (21.8)	
Failure–n(%)	225 (80.6)	139 (82.2)	43 (78.2)	43 (78.2)	
**Median duration of response–months [95%IC]**	9.3 [6.7; 14.0]	9.9 [6.7; 19.2]	6.5 [4.4; 20.8]	13.3 [1; NR]	0.303
**Median OS**^**h**^ **-–months [95%IC]**	10.6 [9.7; 12.1]	12.6 [10.3; 15.6]	7.9 [3.1; 9.8]	10.0 [5.1; 16.4]	<0.001

^a^MDS myelodysplastic syndrome

^b^MPN myeloproliferative neoplasm

^c^WBC white blood cell

^d^LDH lactate deshydrogenase

^e^MRC Medical Research Council

^f^CR complete response

^g^RCi complete response with incomplete hematologic recovery

^h^OS overall survival.

### Prognosis factors for overall survival under azacitidine

Among the 224 patients with available *TP53* status at baseline, we looked for factors affecting overall survival (Table 2). Older age (hazard ratio [HR] = 1.02; 95% CI = [1.00;1.04]; p = 0.040), a higher level of LDH (HR = 1.08; 95% CI = [1.04;1.11]; p<0.001), an adverse karyotype (HR = 1.79; 95% CI = [1.35;2.37]; p<0.001) and presence of *TP53* mutation (HR = 2.22; 95% CI = [1.60;3.08]; p<0.001) or alteration (*TP53* mutation and/or -17/17p-; HR = 2.53; 95% CI = [1.85–3.45]; p<0.001) were significantly associated with a poorer OS in univariable analysis (Table 2).

**Table 2:** Prognosis factors for overall survival in univariable analysis.

	6 mos.-OS (%)	HR [95%CI]	p-value
**Age** (continuous variable)		1.02 [1.00; 1.04]	0.040
**Gender**			
Male	69	1.00	0.299
Female	71	0.86 [0.65; 1.14]	
**AML status**			
De novo	74	1.00	0.558
Secondary	65	1.09 [0.82; 1.43]	
**ECOG Performance status**			
0–1	72	1.00	0.071
2–4	60	1.35 [0.97; 1.86]	
**Charlson score**			
0–1	70	1.00	0.852
>1	72	0.97 [0.68; 1.38]	
**Extramedullary disease**			
No	71	1.00	0.554
Yes	58	1.15 [0.73; 1.81]	
**WBC count** (continuous variable)		1.01 [1.00; 1.02]	0.088
**Platelets count** (continuous variable)		0.87 [0.73; 1.04]	0.117
**LDH** (continuous variable)		1.08 [1.04; 1.11]	<0.001
**Albumin**			
Normal	72	1.00	0.099
< Normal	53	1.42 [0.93; 2.16]	
**Cytogenetic risk (MRC)**			
Non-adverse	82	1.00	<0.001
Adverse	57	1.79 [1.35; 2.37]	
**TP53 mutation**			
No	75	1.00	<0.001
Yes	53	2.22 [1.60; 3.08]	
**TP53 alteration**			
No	79	1.00	<0.001
Yes	49	2.53 [1.85; 3.45]	

In multivariable analysis: age (HR = 1.03; 95% CI = [1.01;1.05]; p = 0.001), LDH (HR = 1.07; 95% CI = [1.03–1.11]; p<0.001), adverse karyotype (HR = 1.58; 95% CI = [1.15–2.34]; p = 0.024) remained significantly associated with OS (Table 3).The effect of *TP53* mutation on OS was just below the threshold of statistical significance (HR = 1.49; 95% CI = [0.95–2.34]; p = 0.081).

**Table 3:** Prognosis factors for overall survival in multivariable analysis.

	HR [95%CI]	*p-value*
**Age** (continuous variable)	1.03 [1.01; 1.06]	*0*.*016*
**ECOG PS**		
0–1	1.00	*0*.*838*
2–4	0.96 [0.67; 1.39]	
**WBC count** (continuous variable)	1.00 [0.99; 1.02]	*0*.*798*
**LDH** (continuous variable/100)	1.07 [1.03; 1.11]	*<0*.*001*
**Platelets count** (continuous variable/100)	0.94 [0.80; 1.09]	*0*.*395*
**Cytogenetic risk**		
Non-adverse	1.00	*0*.*024*
Adverse	1.58 [1.06; 2.34]	
***TP53* mutation**		
No	1.00	*0*.*081*
Yes	1.49 [0.95; 2.34]	

### Patient outcome according to the *TP53* status

**Fig 1:**
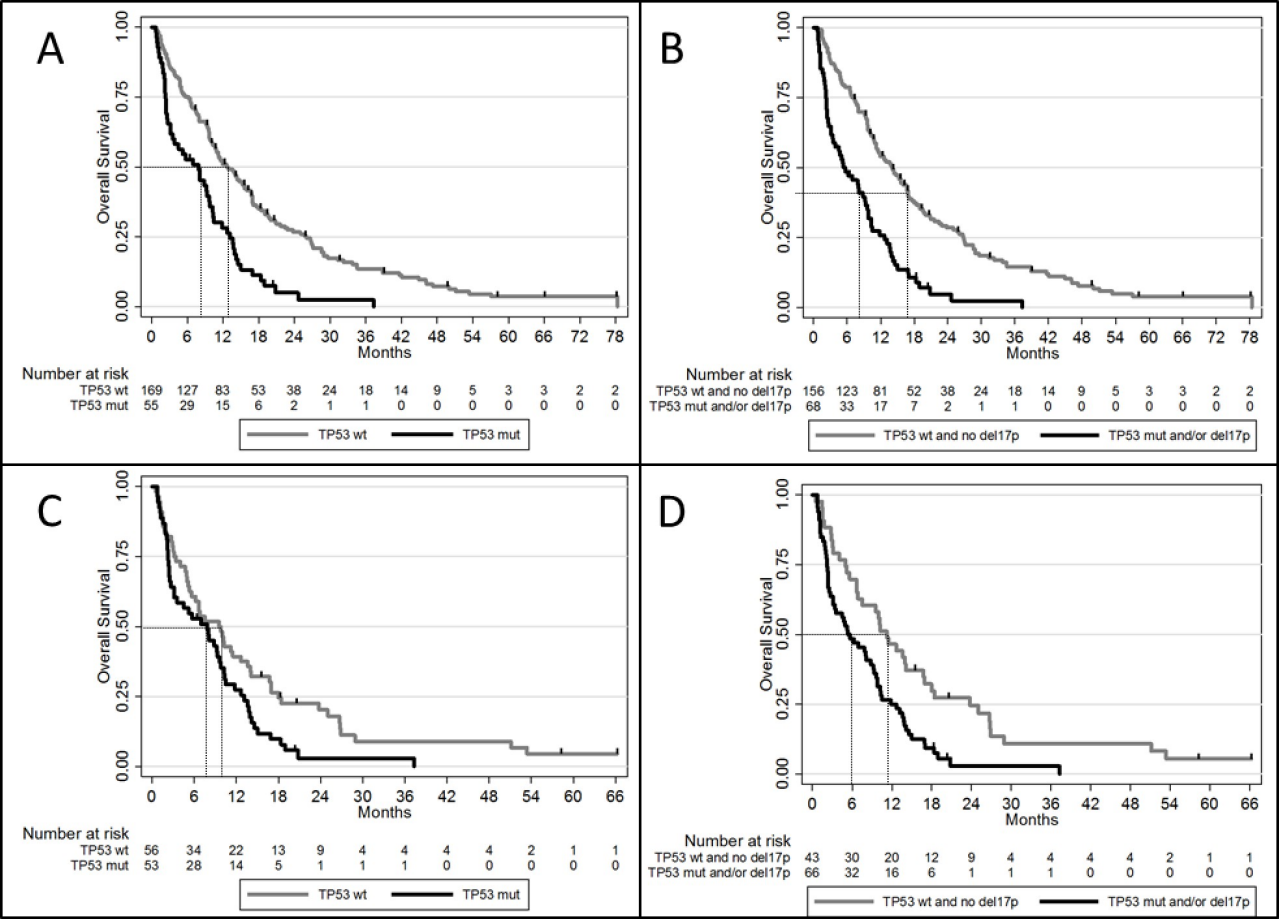
Overall survival according to *TP53* alterations. A. OS in patient with *TP53* mutation versus *TP53*^wt^. B. OS in patient with *TP53* mutation and/or deletion versus patient without TP53 alteration. C. OS in patient with *TP53* mutation versus *TP53*^wt^ in the subgroup of adverse karyotype. D. OS in patient with *TP53* mutation and/or deletion versus patient without *TP53* alteration in the subgroup of adverse karyotype.

The 55 patients with *TP53* mutations had a significantly lower OS compared to wild-type *TP53* patients (Fig 1A; median OS: 7.9 months with *TP53* mutation *vs*. 12.6 months without; HR = 2.22; 95% CI = [1.60–3.08]; p<0.001). The 68 patients with *TP53* alteration (Fig 1B) had a worst OS compared to patients without (median OS: 5.4 vs. 14.0 months, respectively, HR = 2.53; 95% CI = [1.85–3.45]; p<0.001). Within the group of 109 patients with adverse karyotype, *TP53* mutation (Fig 1C; median OS 7.9 months *vs*. 9.6 months, respectively; HR = 1.61; 95% CI = [1.08;2.41]; p = 0.019) and *TP53* alteration including mutation and/or deletion (Fig 1D; median OS 5.4 months *vs*. 11.2 months, respectively, HR = 2.03; 95% CI = [1.33–3.09]; p<0.001) remained significantly associated with poorer OS compared to patients with unaltered *TP53*.

In contrast to OS, response rates (CR/CRi) did not significantly differ according to the presence of *TP53* mutation (21.8% with *TP53* mutations *vs*. 17.8% without; p = 0.502) or alteration *(*19.1% with *vs*. 18.6% without; p = 0.926). Extending the definition of clinical response to partial responses (PR) or hematologic improvement (HI) did not modify the impact of *TP53* mutation in response to azacitidine (CR, CRi and PR: 23.6% with *vs*. 24.3% without; p = 0.925; CR, CRi, PR and HI: 36.4% with *vs*. 42.6% without; p = 0.414). Similarly, within the group of 109 patients with adverse karyotype, *TP53* mutation did not impact response achievement (20.8% RC/RCi with *TP53* mutation *vs*. 14.3% without; p = 0.374).

### Patient outcome according to TP53 mutation classifications

Among the 55 patients with a *TP53* mutation, we identified 49 cases (89%) with a unique *TP53* mutation (42 missenses [86%], 3 nonsenses [6%], and 4 frameshifts [8%]) and 6 cases (11%) with 2 mutations (2 patients with missense and frameshift mutations and 4 with 2 missense mutations).

As the impact of the *TP53* mutations is heterogeneous, we may assume that a specific subgroup of variants might be sensitive to HMA. We evaluated their impact on azacitidine response using three recent classifications of TP53 mutations [24, 25, 33]. Twelve of these patients were classified as responders and 43 as non-responders.

**Fig 2:**
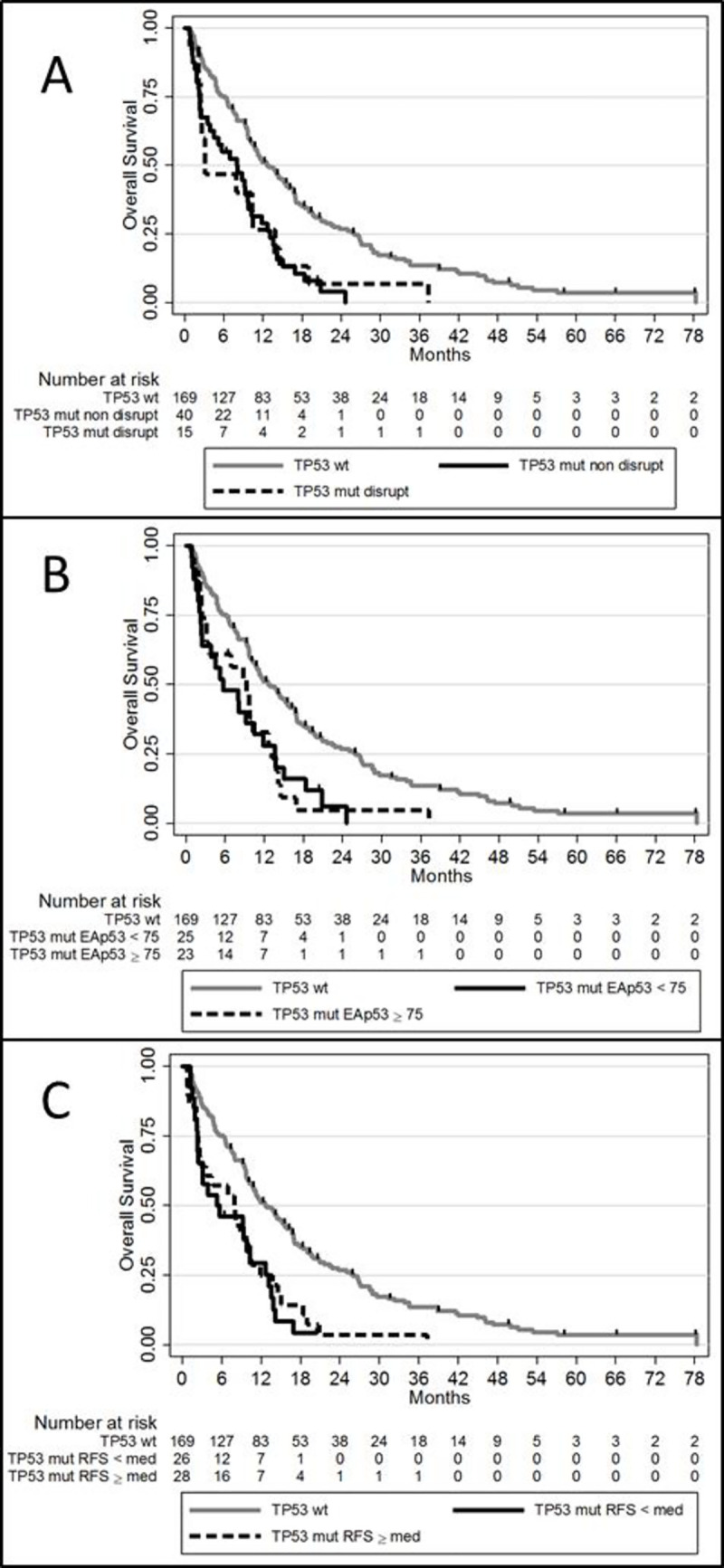
Overall survival according to TP53 mutations classifications. A. OS according to *TP53* disruptive classification: disruptive *TP53* mutation versus non-disruptive *TP53* mutation versus *TP53*^wt^. B. OS according to evolutionary action score: EAp53 < 75 versus EAp53≥ 75 versus *TP53*^wt^. C. OS according to relative flexible score: RFS < median versus ≥ median versus *TP53*^wt^.

Disruptive mutations were detected in 15 patients (27.3%), classification based on the consequences of the *TP53* mutations relying on the location of the mutation and the predicted amino acid alterations. A TP53 Evolutionary Action Score was assessable for 48 patients (87%) and a relative flexible score derived from Kotler *et al*. for 54 patients (98.2%). Functional categorization of *TP53* variant is summarized in S2 Table. Comparison of these 3 different classifications of *TP53* mutations is summarized in Tables 4 and 5. None of these classifications were associated with response to azacitidine or OS (Fig 2).

**Table 4:** Univariable comparison of TP53 mutation functional characterization and response to azacitidine.

	Overall sample n = 55	Responders N = 12	Non responders N = 43	p-value
**Disruptive *TP53* mutation**				
Yes—n(%)	15 (27.3)	3 (25.0)	12 (27.9)	1.000
No—n(%)	40 (72.7)	9 (75.0)	31 (72.1)	
**Evolutionary Action score (n = 48)**,				
continuous variable 0–100	73.9 (28.1–95.7)	79.3 (28.1–89.8)	73.3 (48.5–95.7)	1.000
Median (range)				
**Evolutionary Action score**				
<75—n (%)	25 (52.1)	5 (45.5)	20 (54.1)	0.616
≥75—n (%)	23 (47.9)	6 (54.5)	17 (45.9)	
Missing	7	1	6	
**Relative Fitness score (n = 54)**, log2 scale				0.675
Median (range)	0.094(-2.525–0.838)	0.094(-0.789–0.579)	0.094(-2.525–0.838)	

**Table 5:** Univariable comparison of *TP53* mutation functional characterization and overall survival.

	Event/N	6 mos.-OS (%)	HR [IC95%]	p-value
**Disruptive TP53 mutation**				
No	38/40	55	1.00	0.798
Yes	15/15	47	0.92 [0.50; 1.71]	
**Evolutionary Action score (0–100)**				
<75	24/25	48	1.00	0.923
≥75	22/23	61	0.97 [0.54; 1.75]	
**Evolutionary Action score** (continuous variable, 0–100)			1.01 [0.99; 1.03]	0.515
**Relative Fitness score** log2 scale (continuous)			0.75 [0.45; 1.22]	0.244

## Discussion

Our study of 224 patients constitutes, to our knowledge, the largest cohort of elderly AML patients treated with HMA analyzed for *TP53* mutations so far. We identified an overall prevalence of 24.6%. of *TP53* mutations with a VAF >10%. Among them, 30% were localized in the main *TP53* hotspots for single base substitutions, which is in line with previous descriptions in solid tumors [30] and in AML [12]. The percentage of patients with *TP53* mutation in our cohort is higher than previously reported in elderly AML patients [36–38], which could be easily explained by the high proportion of adverse cytogenetics and secondary AML in this group of patients deemed unfit for IC.

The increasing knowledge of mutant forms of *TP53*, has provided detailed insights into the functional consequences of *TP53* mutations and supports the hypothesis that all *TP53* mutations are not functionally equivalent [23]. The majority of these mutations are missense mutations in the DBD and therefore lead to loss of target gene transactivation [39]. In addition to this loss of function, mutant TP53 may exhibit dominant negative effect on wild-type *TP53* [31] or gain-of-function properties with capacity of transactivating non-canonical target genes that confer selective growth advantage, migratory potential, and drug resistance [40]. Different approaches have been used to systematically categorize various mutant *TP53* forms, based on their functionality in tumor suppression. We selected 3 different classification systems [24, 25, 33] able to characterize *TP53* mutations and we compared this predicted phenotype with patient outcome under azacitidine. Although none of these predictive methods succeed in identifying *TP53* mutated AML patient who could benefit from azacitidine, it remains unknown whether this lack of reliability could be explained by the classification system, or by the biology of this subgroup of AML. It also remains unknown whether these classifications could distinguish *TP53* mutated AML patients with specific outcome treated with other therapy. With the potential differential effect of *TP53* status regarding decitabine or azacitidine therapy, it would be of great interest to investigate the accuracy of these phenotype-genotype tools in predicting the outcome of AML patients treated with decitabine. Given the difficulty of choice between intensive and low-intensity therapy one might also investigate the impact of these TP53 mutant classification system in patients treated with IC.

We did not find any association between *TP53* alterations (including mutation and/or deletion) and response to azacitidine but an association with shorter OS which was significant in univariable analysis (12.6 months in *TP53*wt *versus* 7.9 months in *TP53*mut [p<0.001]] and just below the threshold of significance in multivariable analysis (HR = 1.49; 95% CI = [0.95–2.34]; p = 0.081). This finding is comparable to recent data from phase II trial testing frontline decitabine in AML deemed unfit for IC [7, 41]. Survival outcomes in our cohort are also in line with the biomarker cohort of the phase 3 trial AZA-AML-001 [22], which have a median OS of 7.2 months in *TP53*mut patients compared to 12 months in *TP53*wt patients. This confirms that *TP53* mutations have limited impact on remission achievement in AML as in high-risk MDS but strongly affect OS [42, 43]. We could not reproduce results from Welch *et al*. [21] who reported *TP53* mutation as a positive prognosis factor for response to decitabine without survival advantage, raising the question whether decitabine should be preferred to azacitidine in *TP53* mutated AML patients. We assessed the impact of *TP53* mutation on response rate as defined by CR/CRi, while Welch *et al*. compared *TP53* mutational status and response defined by morphological leukemia free state (MLFS) rate after the first treatment cycle but it was not clear in their description of responders whether patient achieving MLFS after first cycle of decitabine eventually converted to a complete remission or improved OS. Of note, we did not find any impact of *TP53* mutation on overall response rate when response was defined with less stringent criteria including patients achieving at least HI. The differences could also rely on the doses of HMA administrated, Welch *et al*. used decitabine at a 200mg/m^2^ divided daily dose for 10 days every 4 weeks, i.e. twice the dose of the current FDA approved scheme for high risk MDS and more than twice the monthly dose of the regimen tested by Lübbert *et al*. in elderly patients with AML [7]. Although, Blum *et al*. [44] reported improved CR rate in a phase II trial of elderly AML patients with this intensified scheme, the preferred dose and schedule of decitabine remains uncertain and is mainly limited by the myelotoxicity of the drug. Even though decitabine and azacitidine are both cytosine analogs with identical ring structure, they differ by the sugar attached to this ring. The deoxyribose in decitabine allows the incorporation of all metabolites to DNA, whereas only 10–20% of azacitidine is converted into a deoxyribonucleotide, the remaining of the drug being incorporated into RNA. The mechanism of action of both drugs is not fully understood and observed differences in outcome with decitabine compared to azacitidine for patient with specific genotype could presumably give information into precise mechanisms of action of these drugs.

In depth *TP53* genetic integrity analysis will also become inevitable for patients treated with FDA-approved association of HMA and BCL-2 inhibitor venetoclax. Recent data on molecular predictors of response with venetoclax combinations in older patients with AML indicate that TP53 loss promotes resistance to both venetoclax and chemotherapy with apparition of biallelic TP53 defectives clones at progression [45]. It remains unknown if a subset of TP53 abnormalities evase this selective pressure.

Regarding the growing field of *TP53*-activating compounds [46] and targeted therapy against *TP53* pathway genes [47] (*e*.*g*., *MDM2*), a better characterization of mutational and non-mutational TP53 alterations will become useful in the initial workup of each AML patient [48]. In this regard, our cohort constitutes a reference for ongoing non-randomized phase II trial testing these *TP53*-activating compounds whose results are eagerly anticipated.

## Supporting information

S1 Table Primers used for TP53 targeted sequencing. (DOCX)

S2 Table *TP53* mutation functional characterization and patient outcome. (DOCX)

S1 Fig Overall survival according to *TP53* mutation with a threshold ≥1%. (DOCX)

## References

[1] Bertoli S, Tavitian S, Huynh A, Borel C, Guenounou S, Luquet I, et al Improved outcome for AML patients over the years 2000–2014. Blood Cancer J. 2017 11 29;7(12):635 10.1038/s41408-017-0011-1 29184070 PMC5802565

[2] Derolf AR, Kristinsson SY, Andersson TM-L, Landgren O, Dickman PW, Björkholm M. Improved patient survival for acute myeloid leukemia: a population-based study of 9729 patients diagnosed in Sweden between 1973 and 2005. Blood. 2009 4 16;113(16):3666–72. 10.1182/blood-2008-09-179341 19020306

[3] Juliusson G, Swedish AML Group. Most 70- to 79-year-old patients with acute myeloid leukemia do benefit from intensive treatment. Blood. 2011 3 24;117(12):3473–4. 10.1182/blood-2010-11-321737 21436081

[4] Kantarjian H, Ravandi F, O’Brien S, Cortes J, Faderl S, Garcia-Manero G, et al Intensive chemotherapy does not benefit most older patients (age 70 years or older) with acute myeloid leukemia. Blood. 2010 11 25;116(22):4422–9. 10.1182/blood-2010-03-276485 20668231 PMC4081299

[5] Bories P, Lamy S, Simand C, Bertoli S, Delpierre C, Malak S, et al Physician uncertainty aversion impacts medical decision making for older patients with acute myeloid leukemia: results of a national survey. Haematologica. 2018;103(12):2040–8. 10.3324/haematol.2018.192468 30006448 PMC6269286

[6] Dombret H, Seymour JF, Butrym A, Wierzbowska A, Selleslag D, Jang JH, et al International phase 3 study of azacitidine vs conventional care regimens in older patients with newly diagnosed AML with >30% blasts. Blood. 2015 7 16;126(3):291–9. 10.1182/blood-2015-01-621664 25987659 PMC4504945

[7] Lübbert M, Rüter BH, Claus R, Schmoor C, Schmid M, Germing U, et al A multicenter phase II trial of decitabine as first-line treatment for older patients with acute myeloid leukemia judged unfit for induction chemotherapy. Haematologica. 2012 3;97(3):393–401. 10.3324/haematol.2011.048231 22058219 PMC3291594

[8] Ravandi F, Issa J-P, Garcia-Manero G, O’Brien S, Pierce S, Shan J, et al Superior outcome with hypomethylating therapy in patients with acute myeloid leukemia and high-risk myelodysplastic syndrome and chromosome 5 and 7 abnormalities. Cancer. 2009 12 15;115(24):5746–51. 10.1002/cncr.24661 19795507 PMC2794953

[9] Ritchie EK, Feldman EJ, Christos PJ, Rohan SD, Lagassa CB, Ippoliti C, et al Decitabine in patients with newly diagnosed and relapsed acute myeloid leukemia. Leuk Lymphoma. 2013 9;54(9):2003–7. 10.3109/10428194.2012.762093 23270581 PMC3888021

[10] Bowen D, Groves MJ, Burnett AK, Patel Y, Allen C, Green C, et al TP53 gene mutation is frequent in patients with acute myeloid leukemia and complex karyotype, and is associated with very poor prognosis. Leukemia. 2009 1;23(1):203–6. 10.1038/leu.2008.173 18596741

[11] Haferlach C, Dicker F, Herholz H, Schnittger S, Kern W, Haferlach T. Mutations of the TP53 gene in acute myeloid leukemia are strongly associated with a complex aberrant karyotype. Leukemia. 2008 8;22(8):1539–41. 10.1038/leu.2008.143 18528419

[12] Rücker FG, Schlenk RF, Bullinger L, Kayser S, Teleanu V, Kett H, et al TP53 alterations in acute myeloid leukemia with complex karyotype correlate with specific copy number alterations, monosomal karyotype, and dismal outcome. Blood. 2012 3 1;119(9):2114–21. 10.1182/blood-2011-08-375758 22186996

[13] Kadia TM, Jain P, Ravandi F, Garcia-Manero G, Andreef M, Takahashi K, et al TP53 mutations in newly diagnosed acute myeloid leukemia: Clinicomolecular characteristics, response to therapy, and outcomes. Cancer. 2016 11 15;122(22):3484–91. 10.1002/cncr.30203 27463065 PMC5269552

[14] Middeke JM, Herold S, Rücker-Braun E, Berdel WE, Stelljes M, Kaufmann M, et al TP53 mutation in patients with high-risk acute myeloid leukaemia treated with allogeneic haematopoietic stem cell transplantation. Br J Haematol. 2016 3;172(6):914–22. 10.1111/bjh.13912 26771088

[15] Ciurea SO, Chilkulwar A, Saliba RM, Chen J, Rondon G, Patel KP, et al Prognostic factors influencing survival after allogeneic transplantation for AML/MDS patients with TP53 mutations. Blood. 2018 6 28;131(26):2989–92. 10.1182/blood-2018-02-832360 29769261 PMC7218750

[16] Bejar R, Stevenson KE, Caughey B, Lindsley RC, Mar BG, Stojanov P, et al Somatic mutations predict poor outcome in patients with myelodysplastic syndrome after hematopoietic stem-cell transplantation. J Clin Oncol. 2014 9 1;32(25):2691–8. 10.1200/JCO.2013.52.3381 25092778 PMC4207878

[17] Nieto M, Samper E, Fraga MF, González de Buitrago G, Esteller M, Serrano M. The absence of p53 is critical for the induction of apoptosis by 5-aza-2’-deoxycytidine. Oncogene. 2004 1 22;23(3):735–43. 10.1038/sj.onc.1207175 14737108

[18] Yi L, Sun Y, Levine A. Selected drugs that inhibit DNA methylation can preferentially kill p53 deficient cells. Oncotarget. 2014 10 15;5(19):8924–36. 10.18632/oncotarget.2441 25238040 PMC4253407

[19] McKay BC, Becerril C, Ljungman M. P53 plays a protective role against UV- and cisplatin-induced apoptosis in transcription-coupled repair proficient fibroblasts. Oncogene. 2001 10 11;20(46):6805–8. 10.1038/sj.onc.1204901 11709715

[20] Gudkov AV, Komarova EA. The role of p53 in determining sensitivity to radiotherapy. Nat Rev Cancer. 2003 2;3(2):117–29. 10.1038/nrc992 12563311

[21] Welch JS, Petti AA, Miller CA, Fronick CC, O’Laughlin M, Fulton RS, et al TP53 and Decitabine in Acute Myeloid Leukemia and Myelodysplastic Syndromes. N Engl J Med. 2016 24;375(21):2023–36. 10.1056/NEJMoa1605949 27959731 PMC5217532

[22] Döhner H, Dolnik A, Tang L, Seymour JF, Minden MD, Stone RM, et al Cytogenetics and gene mutations influence survival in older patients with acute myeloid leukemia treated with azacitidine or conventional care. Leukemia. 2018 12;32(12):2546–57. 10.1038/s41375-018-0257-z 30275526 PMC6286388

[23] Sabapathy K, Lane DP. Therapeutic targeting of p53: all mutants are equal, but some mutants are more equal than others. Nat Rev Clin Oncol. 2018 1;15(1):13–30. 10.1038/nrclinonc.2017.151 28948977

[24] Poeta ML, Manola J, Goldwasser MA, Forastiere A, Benoit N, Califano JA, et al TP53 mutations and survival in squamous-cell carcinoma of the head and neck. N Engl J Med. 2007 12 20;357(25):2552–61. 10.1056/NEJMoa073770 18094376 PMC2263014

[25] Neskey DM, Osman AA, Ow TJ, Katsonis P, McDonald T, Hicks SC, et al Evolutionary Action Score of TP53 Identifies High-Risk Mutations Associated with Decreased Survival and Increased Distant Metastases in Head and Neck Cancer. Cancer Res. 2015 4 1;75(7):1527–36. 10.1158/0008-5472.CAN-14-2735 25634208 PMC4383697

[26] Young KH, Weisenburger DD, Dave BJ, Smith L, Sanger W, Iqbal J, et al Mutations in the DNA-binding codons of TP53, which are associated with decreased expression of TRAILreceptor-2, predict for poor survival in diffuse large B-cell lymphoma. Blood. 2007 12 15;110(13):4396–405. 10.1182/blood-2007-02-072082 17881637 PMC2234786

[27] Bories P, Bertoli S, Bérard E, Laurent J, Duchayne E, Sarry A, et al Intensive chemotherapy, azacitidine, or supportive care in older acute myeloid leukemia patients: an analysis from a regional healthcare network. Am J Hematol. 2014 12;89(12):E244–252. 10.1002/ajh.23848 25195872

[28] Dumas P-Y, Bertoli S, Bérard E, Médiavilla C, Yon E, Tavitian S, et al Azacitidine or intensive chemotherapy for older patients with secondary or therapy-related acute myeloid leukemia. Oncotarget. 2017 10 3;8(45):79126–36. 10.18632/oncotarget.15988 29108292 PMC5668025

[29] Grimwade D, Hills RK, Moorman AV, Walker H, Chatters S, Goldstone AH, et al Refinement of cytogenetic classification in acute myeloid leukemia: determination of prognostic significance of rare recurring chromosomal abnormalities among 5876 younger adult patients treated in the United Kingdom Medical Research Council trials. Blood. 2010 7 22;116(3):354–65. 10.1182/blood-2009-11-254441 20385793

[30] Bouaoun L, Sonkin D, Ardin M, Hollstein M, Byrnes G, Zavadil J, et al TP53 Variations in Human Cancers: New Lessons from the IARC TP53 Database and Genomics Data. Hum Mutat. 2016;37(9):865–76. 10.1002/humu.23035 27328919

[31] Willis A, Jung EJ, Wakefield T, Chen X. Mutant p53 exerts a dominant negative effect by preventing wild-type p53 from binding to the promoter of its target genes. Oncogene. 2004 3 25;23(13):2330–8. 10.1038/sj.onc.1207396 14743206

[32] Chun YS, Passot G, Yamashita S, Nusrat M, Katsonis P, Loree JM, et al Deleterious Effect of RAS and Evolutionary High-risk TP53 Double Mutation in Colorectal Liver Metastases. Ann Surg. 2019;269(5):917–23. 10.1097/SLA.0000000000002450 28767562 PMC7462436

[33] Kotler E, Shani O, Goldfeld G, Lotan-Pompan M, Tarcic O, Gershoni A, et al A Systematic p53 Mutation Library Links Differential Functional Impact to Cancer Mutation Pattern and Evolutionary Conservation. Mol Cell. 2018 7 5;71(1):178–190.e8. 10.1016/j.molcel.2018.06.012 29979965

[34] Döhner H, Estey E, Grimwade D, Amadori S, Appelbaum FR, Büchner T, et al Diagnosis and management of AML in adults: 2017 ELN recommendations from an international expert panel. Blood. 2017 1 26;129(4):424–47. 10.1182/blood-2016-08-733196 27895058 PMC5291965

[35] Cheson BD, Greenberg PL, Bennett JM, Lowenberg B, Wijermans PW, Nimer SD, et al Clinical application and proposal for modification of the International Working Group (IWG) response criteria in myelodysplasia. Blood. 2006 7 15;108(2):419–25. 10.1182/blood-2005-10-4149 16609072

[36] Stengel A, Kern W, Haferlach T, Meggendorfer M, Fasan A, Haferlach C. The impact of TP53 mutations and TP53 deletions on survival varies between AML, ALL, MDS and CLL: an analysis of 3307 cases. Leukemia. 2017;31(3):705–11. 10.1038/leu.2016.263 27680515

[37] Tsai C-H, Hou H-A, Tang J-L, Liu C-Y, Lin C-C, Chou W-C, et al Genetic alterations and their clinical implications in older patients with acute myeloid leukemia. Leukemia. 2016 7;30(7):1485–92. 10.1038/leu.2016.65 27055875

[38] Prassek VV, Rothenberg-Thurley M, Sauerland MC, Herold T, Janke H, Ksienzyk B, et al Genetics of acute myeloid leukemia in the elderly: mutation spectrum and clinical impact in intensively treated patients aged 75 years or older. Haematologica. 2018;103(11):1853–61. 10.3324/haematol.2018.191536 29903761 PMC6278991

[39] Lee MK, Teoh WW, Phang BH, Tong WM, Wang ZQ, Sabapathy K. Cell-type, dose, and mutation-type specificity dictate mutant p53 functions in vivo. Cancer Cell. 2012 12 11;22(6):751–64. 10.1016/j.ccr.2012.10.022 23238012

[40] Blandino G, Levine AJ, Oren M. Mutant p53 gain of function: differential effects of different p53 mutants on resistance of cultured cells to chemotherapy. Oncogene. 1999 1 14;18(2):477–85. 10.1038/sj.onc.1202314 9927204

[41] Becker H, Pfeifer D, Ihorst G, Pantic M, Wehrle J, Rüter BH, et al Monosomal karyotype and chromosome 17p loss or TP53 mutations in decitabine-treated patients with acute myeloid leukemia. Ann Hematol. 2020 7;99(7):1551–60. 10.1007/s00277-020-04082-7 32504186 PMC7316846

[42] Bejar R, Lord A, Stevenson K, Bar-Natan M, Pérez-Ladaga A, Zaneveld J, et al TET2 mutations predict response to hypomethylating agents in myelodysplastic syndrome patients. Blood. 2014 10 23;124(17):2705–12. 10.1182/blood-2014-06-582809 25224413 PMC4208285

[43] Bally C, Adès L, Renneville A, Sebert M, Eclache V, Preudhomme C, et al Prognostic value of TP53 gene mutations in myelodysplastic syndromes and acute myeloid leukemia treated with azacitidine. Leuk Res. 2014 7;38(7):751–5. 10.1016/j.leukres.2014.03.012 24836762

[44] Blum W, Garzon R, Klisovic RB, Schwind S, Walker A, Geyer S, et al Clinical response and miR-29b predictive significance in older AML patients treated with a 10-day schedule of decitabine. Proc Natl Acad Sci USA. 2010 4 20;107(16):7473–8. 10.1073/pnas.1002650107 20368434 PMC2867720

[45] DiNardo CD, Tiong IS, Quaglieri A, MacRaild S, Loghavi S, Brown FC, et al Molecular patterns of response and treatment failure after frontline venetoclax combinations in older patients with AML. Blood. 2020 3 12;135(11):791–803. 10.1182/blood.2019003988 31932844 PMC7068032

[46] Bykov VJN, Wiman KG. Mutant p53 reactivation by small molecules makes its way to the clinic. FEBS Lett. 2014 8 19;588(16):2622–7. 10.1016/j.febslet.2014.04.017 24768524

[47] Andreeff M, Kelly KR, Yee K, Assouline S, Strair R, Popplewell L, et al Results of the Phase I Trial of RG7112, a Small-Molecule MDM2 Antagonist in Leukemia. Clin Cancer Res. 2016 2 15;22(4):868–76. 10.1158/1078-0432.CCR-15-0481 26459177 PMC4809642

[48] Prokocimer M, Molchadsky A, Rotter V. Dysfunctional diversity of p53 proteins in adult acute myeloid leukemia: projections on diagnostic workup and therapy. Blood. 2017 10;130(6):699–712. 10.1182/blood-2017-02-763086 28607134 PMC5659817

