# Supplementary material for: Impact of TP53 mutations in acute myeloid leukemia patients treated with azacitidine
Source: PLoS One. 2020 Oct 1;15(10):e0238795. doi: 10.1371/journal.pone.0238795 (PMC7529302; doi:10.1371/journal.pone.0238795)
Supplement: S1 Table — (DOCX) [file pone.0238795.s001.docx]

**S1 Table: Primers used for TP53 targeted sequencing**

| **Exon** | **Primer** |
| --- | --- |
| TP53_X04_F3 | TCGTCGGCAGCGTCAGATGTGTATAAGAGACAGCTGCACCAGCAGCTCCTACA |
| TP53_X04_R22 | GTCTCGTGGGCTCGGAGATGTGTATAAGAGACAGCAGGCATTGAAGTCTCATGGAA |
| TP53_X05_F12 | TCGTCGGCAGCGTCAGATGTGTATAAGAGACAGCAACTCTGTCTCCTTCCTCTTCCTAC |
| TP53_X05_R22 | GTCTCGTGGGCTCGGAGATGTGTATAAGAGACAGGTCGTCTCTCCAGCCCCAGC |
| TP53_X06_F1 | TCGTCGGCAGCGTCAGATGTGTATAAGAGACAGCCTCTGATTCCTCACTGATTGC |
| TP53_X06_R1 | GTCTCGTGGGCTCGGAGATGTGTATAAGAGACAGCTTAACCCCTCCTCCCAGAG |
| TP53_X07_F1 | TCGTCGGCAGCGTCAGATGTGTATAAGAGACAGTTGGGCCTGTGTTATCTCCT |
| TP53_X07_R1 | GTCTCGTGGGCTCGGAGATGTGTATAAGAGACAGTGGCAAGTGGCTCCTGAC |
| TP53_X08_F1 | TCGTCGGCAGCGTCAGATGTGTATAAGAGACAGTTGCTTCTCTTTTCCTATCCTGA |
| TP53_X08_R1 | GTCTCGTGGGCTCGGAGATGTGTATAAGAGACAGGCTTCTTGTCCTGCTTGCTT |
| TP53_X09_F1 | TCGTCGGCAGCGTCAGATGTGTATAAGAGACAGCCTTTCCTTGCCTCTTTCCT |
| TP53_X09_R1 | GTCTCGTGGGCTCGGAGATGTGTATAAGAGACAGCCACTTGATAAGAGGTCCCAAG |
| TP53_X10_F2 | TCGTCGGCAGCGTCAGATGTGTATAAGAGACAGGAACCATCTTTTAACTCAGGTACTGTGTAT |
| TP53_X10_R1 | GTCTCGTGGGCTCGGAGATGTGTATAAGAGACAGGAAGGGGCTGAGGTCACTC |
