# Supplementary material for: Impact of TP53 mutations in acute myeloid leukemia patients treated with azacitidine
Source: PLoS One. 2020 Oct 1;15(10):e0238795. doi: 10.1371/journal.pone.0238795 (PMC7529302; doi:10.1371/journal.pone.0238795)
Supplement: S2 Table — (DOCX) [file pone.0238795.s002.docx]

| **Case** | **Codon** | **AA change** | **Disruptive mutation** | **EA p53 score** | **RFS (log2)** | **Response** | **Follow up status (months)** |
| --- | --- | --- | --- | --- | --- | --- | --- |
| 11 | 154 | Gly>Val | Non-disruptive | 89.76 | -0.7895 | CRi | Dead(9.9) |
| 15 | 248 | Arg>Trp | Disruptive | 84.11 | 0.0740 | CR | Dead(14) |
| 17 | 103 | Tyr>FS | Disruptive | - | 0.2865 | Failure | Dead(2.7) |
| 20 | 282 | Arg>Trp | Non-disruptive | 73.21 | 0.0160 | Failure | Dead(1.9) |
| 21 | 143 | Val>M | Non-disruptive | 51.72 | -1.3307 | Failure | Dead(1.2) |
| 25 | 248 | Arg>Trp | Disruptive | 84.11 | 0.0740 | Failure | Dead(3.1) |
| 31 | 280 | Arg>Gly | Non-disruptive | 95.71 | -0.5223 | Failure | Dead(1.3) |
| 34 | 273 | Arg>His | Non-disruptive | 66.12 | 0.2503 | Failure | Dead(0.9) |
| 37 | 234 | Tyr>Cys | Disruptive | - | 0.7908 | Failure | Dead(10.6) |
| 42 | 220 | Tyr>Cys | Non-disruptive | 72.52 | 0.0944 | CR | Dead(18.5) |
| 48 | 175 | Arg>His | Non-disruptive | 78.51 | -0.1449 | Failure | Dead(2.2) |
| 55 | 250 | Pro>Leu | Non-disruptive | 89 | -0.2583 | Failure | Dead(9.4) |
| 56 | 276 | Ala>Gly | Non-disruptive | 60 | -0.0626 | Failure | Dead(9.3) |
| 58 | 59 | Pro>FS | Disruptive | - | 0,1452 | CR | Dead(19.1) |
| 69 | 220 | Tyr>Cys | Non-disruptive | 72.52 | 0.0943 | HI | Dead(9.7) |
| 70 | 91 | Trp>X | Disruptive | 48.46 | 0.8378 | Failure | Dead(2.1) |
| 90 | 158 | Arg>His | Non-disruptive | 80.98 | -0.5980 | CR | Dead(12.8) |
| 94 | 273 | Arg>His | Non-disruptive | 66.12 | 0.2503 | Failure | Dead(4.6) |
| 101 | 273 | Arg>Cys | Non-disruptive | 84.52 | -0.1254 | Failure | Dead(2.4) |
| 104 | 360 | Gly>Ala | Non-disruptive | 28.06 |  | CR | Dead(24.8) |
| 109 | 179 | His>Tyr | Disruptive | 77.78 | 0.6499 | PR | Dead(14.7) |
| 111 | 273 | Arg>His | Non-disruptive | 66.12 | 0.2503 | HI | Dead(11.9) |
| 123 | 144 | Gln>FS | Disruptive |  | 0.4349 | Failure | Dead(2.6) |
| 124 | 232 | Ile>Ser | Non-disruptive | 67.76 | -0.0234 | CR | Alive (20.4) |
| 130 | 273 | Arg>Cys | Non-disruptive | 84.52 | -0.1254 | CR | Dead(14.3) |
| 133 | 236 | Tyr>Cys | Non-disruptive | 62.93 | 0.0888 | HI | Dead(13.7) |
| 135 | 216 | Val>Met | Non-disruptive | 73.3 | -1.9189 | Failure | Dead(2.3) |
| 138 | 253 | Thr>Ala | Disruptive | 89.56 | -0.2070 | Failure | Dead(2.5) |
| 139 | 273 | Arg>His | Non-disruptive | 66.12 | 0.2503 | Failure | Dead(8.2) |
| 140 | 275 | Cys>Tyr | Non-disruptive | 93.47 | 0.4055 | HI | Dead(7) |
| 152 | 135 | Cys>Tyr | Non-disruptive | 79.31 | 0.1868 | CRi | Dead(8.9) |
| 154 | 272 | Val>Met | Non-disruptive | 63.49 | 0.2274 | CR | Dead(15.1) |
| 158 | 127 | Ser>Thr | Non-disruptive | 64 | 0.5790 | CR | Dead(13.9) |
| 160 | 273 | Arg>Leu | Non-disruptive | 87.45 | 0.0445 | Failure | Dead(13.2) |
| 161 | 238 | Cys>Tyr | Non-disruptive | 92.66 | 0.2148 | Failure | Dead(0.8) |
| 163 | 245 | Gly>Ser | Non-disruptive | 89.56 | 0.2621 | Failure | Dead(3.6) |
| 174 | 215 | Ser>Arg | Non-disruptive | 89.07 | 0.0033 | Failure | Dead(1.9) |
| 181 | 136 | Gln>X | Disruptive | - | 0.1405 | Failure | Dead(10.4) |
| 182 | 136 | Gln>Glu | Non-disruptive | 50.94 | -0.0856 | Failure | Dead(1.6) |
| 189 | 136 | Gln>Glu | Non-disruptive | 50.94 | -0.0856 | HI | Dead(5.7) |
| 195 | 241 | Ser>FS | Disruptive | - | 0.5295 | Failure | Dead(7.9) |
| 197 | 220 | Tyr>Asn | Non-disruptive | 74.47 | 0.1565 | Failure | Dead(1) |
| 200 | 150 | Thr>FS | Disruptive | - | 0,3863 | Failure | Dead(2.2) |
| 202 | 248 | Arg>His | Disruptive | 84.11 | 0.0739 | CR | Dead(37.6) |
| 217 | 175 | Arg>His | Non-disruptive | 78.51 | -0.1449 | Failure | Dead(10.4) |
| 227 | 213 | Arg>X | Disruptive | - | 0.5642 | Failure | Dead(0.8) |
| 240 | 283 | Arg>Cys | Non-disruptive | 69.69 | -1.5004 | Failure | Dead(3.9) |
| 243 | 135 | Cys>Thr | Non-disruptive | 69.53 | -2.5250 | Failure | Dead(5.3) |
| 248 | 220 | Tyr>Cys | Non-disruptive | 72.52 | 0.0944 | HI | Dead(21) |
| 249 | 280 | Arg>Lys | Non-disruptive | 71.18 | 0.1868 | Failure | Dead(8.1) |
| 251 | 215 | Ser>Asn | Non-disruptive | 68.04 | 0.2742 | Failure | Dead(2.4) |
| 269 | 273 | Arg>Cys | Non-disruptive | 84.52 | -0.1254 | Failure | Alive (6.3) |
| 271 | 234 | Tyr>Cys | Non-disruptive | 62.94 | 0.5111 | Failure | Dead(2.5) |
| 276 | 248 | Arg>Trp | Disruptive | 84.11 | 0.0740 | Failure | Dead(3.1) |
| 285 | 273 | Arg>Ser | Non-disruptive | 85.17 | 0.0550 | HI | Dead(17.1) |

**S2 Table: TP53 mutation functional characterization and patient outcome**

Disruptive mutation affected L2/L3 loops of the DNA binding domain or were truncative mutations, EA TP53 score: evolutionary action score calculated with http://mammoth.bcm.tmc.edu/cgi-bin/panos/EAp53.cgi; RFS: relative flexible score, CR complete remission, CRi complete remission with incomplete hematological recovery, HI hematological improvement.
