# Supplementary material for: Impact of TP53 mutations in acute myeloid leukemia patients treated with azacitidine
Source: PLoS One. 2020 Oct 1;15(10):e0238795. doi: 10.1371/journal.pone.0238795 (PMC7529302; doi:10.1371/journal.pone.0238795)
Supplement: S1 Fig — (DOCX) [file pone.0238795.s003.docx]

# S1 Fig: Overall survival according to *TP53* mutation with a threshold ≥1%
